# Identification of Key Amino Acids in the PB2 and M1 Proteins of H7N9 Influenza Virus That Affect Its Transmission in Guinea Pigs

**DOI:** 10.1128/JVI.01180-19

**Published:** 2019-12-12

**Authors:** Huihui Kong, Shujie Ma, Jingfei Wang, Chunyang Gu, Zeng Wang, Jianzhong Shi, Guohua Deng, Yuntao Guan, Hualan Chen

**Affiliations:** a State Key Laboratory of Veterinary Biotechnology, Harbin Veterinary Research Institute, CAAS, Harbin, People’s Republic of China; University of Southern California

**Keywords:** H7N9, transmissibility, genetic basis

## Abstract

Efficient human-to-human transmission is a prerequisite for a novel influenza virus to cause an influenza pandemic; however, the genetic determinants of influenza virus transmission are still not fully understood. In this study, we compared the respiratory droplet transmissibilities of four H7N9 viruses that are genetic closely related and found that these viruses have dissimilar transmissibilities in guinea pigs: A/Anhui/1/2013 (AH/1) transmitted efficiently, whereas the other three viruses did not transmit. The three nontransmissible viruses have one to eight amino acid differences compared with the AH/1 virus. To investigate which of these amino acids is important for transmission, we used reverse genetics to generate a series of reassortants and mutants in the AH/1 background and tested their transmissibility in guinea pigs. We found that the neuraminidase (NA) of the nontransmissible virus A/chicken/Shanghai/S1053/2013 had low enzymatic activity that impaired the transmission of AH/1 virus, and three amino acid mutations—V292I and K627E in PB2 and D156E in M1—independently abolished the transmission of the AH/1 virus. We further found that an NA reassortant and three single-amino-acid mutants replicated less efficiently than the AH/1 virus in A549 cells and that the amino acid at position 156 of M1 affected the morphology of H7N9 viruses. Our study identifies key amino acids in PB2 and M1 that play important roles in H7N9 inﬂuenza virus transmission and provides new insights into the transmissibility of inﬂuenza virus.

**IMPORTANCE** Efficient transmission is a prerequisite for a novel influenza virus to cause an influenza pandemic; however, the genetic determinants of influenza virus transmission remain poorly understood. H7N9 influenza viruses, which emerged in 2013 in China, have caused over 1,560 human infection cases, showing clear pandemic potential. Previous studies have shown that the H7N9 viruses differ in their transmissibility in animal models. In this study, we found two amino acids in PB2 (292V and 627K) and one in M1 (156D) that are extremely important for H7N9 virus transmission. Of note, PB2 292V and M1 156D appear in most H7N9 viruses, and the PB2 627K mutation could easily occur when the H7N9 virus replicates in humans. Our study thus identifies new amino acids that are important for influenza virus transmission and suggests that just a few key amino acid changes can render the H7N9 virus transmissible in mammals.

## Abstract (precis)

Efficient transmission is a prerequisite for a novel influenza virus to cause an influenza pandemic; however, the genetic determinants of influenza virus transmission remain poorly understood. H7N9 influenza viruses, which emerged in 2013 in China, have caused over 1,560 human infection cases, showing clear pandemic potential. Previous studies have shown that the H7N9 viruses differ in their transmissibility in animal models. In this study, we found two amino acids in PB2 (292V and 627K) and one in M1 (156D) that are extremely important for H7N9 virus transmission. Of note, PB2 292V and M1 156D appear in most H7N9 viruses, and the PB2 627K mutation could easily occur when the H7N9 virus replicates in humans. Our study thus identifies new amino acids that are important for influenza virus transmission and suggests that just a few key amino acid changes can render the H7N9 virus transmissible in mammals.

## INTRODUCTION

Influenza A virus, which belongs to the *Orthomyxoviridae* family, consists of eight negative-sense, single-strand RNA segments encoding as many as 17 proteins (1–6). Influenza A viruses are divided into 18 hemagglutinin (HA) and 11 neuraminidase (NA) subtypes according to the genetic and antigenic variability of these surface proteins. The H17N10 and H18N11 subtypes were identified in bats (7, 8), whereas all other subtypes have been detected in avian species (9, 10). Influenza A viruses are important pathogens that can cause infection and disease in both animals and humans. H5 and H7, highly pathogenic influenza viruses, have caused numerous disease outbreaks in poultry around the world, resulting in tremendous economic losses to the poultry industry. Three subtypes (H1N1, H2N2, and H3N2) of influenza viruses have caused influenza pandemics in humans. Viruses of several other subtypes, including H5N1, H5N6, H7N7, H7N9, H9N2, and H10N8, have crossed the species barrier and caused multiple human infections in different countries (11–16), raising the concern that a new influenza pandemic could occur if any of these avian influenza viruses acquires the ability to transmit efficiently from human to human.

Two animal models, the ferret and guinea pig, have been widely used to evaluate the transmission potential of influenza viruses in humans and to identify key amino acids that contribute to their transmissibility (17–31). Tumpey et al. reported that two amino acid mutations, D190E and D225G, abolished the ability of the 1918/H1N1 virus to transmit via respiratory droplets between ferrets (19). Zhang et al. reported that 226Q in HA is important for the transmission of the 2009/H1N1 pandemic virus in both guinea pigs and ferrets (21). Wang et al. reported that 225E of HA is important for the respiratory droplet transmission of Eurasian avian-like H1N1 swine influenza virus in guinea pigs (20). Gao et al. and Herfst et al. reported that the absence of glycosylation at residues 158 to 160 in the HA of H5N1 viruses is pivotal to their affinity for human-type receptors and transmission in guinea pigs and ferrets (22, 25). Several amino acids in basic polymerase 2 (PB2) that play key roles in the transmission of influenza viruses have also been identified (21, 25, 26, 32). The amino acid mutation A271T in PB2 eliminated the transmission of 2009/H1N1 pandemic virus in guinea pigs (21), and the amino acids 627K and 701N in PB2 increased the transmission of H3N2 virus and H5N1 virus in guinea pigs (25, 26) and the transmission of H7N9 virus and H9N2 virus in ferrets (27, 28). Zhang et al. reported that H5N1 viruses bearing the PA gene or NS gene of the 2009/H1N1 pandemic virus are transmissible in guinea pigs (23). These studies indicate that the transmissibility of influenza virus is a polygenic trait and that more genetic determinants remain to be revealed.

The H7N9 influenza viruses have caused over 1,560 human infections since they emerged in China in 2013 (33–35). We previously evaluated five H7N9 influenza viruses and found that their transmissibilities in ferrets differ: one human isolate, A/Anhui/1/2013 (AH/1), transmitted to all of three exposed ferrets, one avian isolate, A/chicken/Shanghai/S1053/2013 (CK/S1053), did not transmit, and each of the other three viruses transmitted to only one of the three exposed ferrets (29). In the present study, we evaluated the transmissibilities of four genetically similar H7N9 viruses in guinea pigs and found that the AH/1 virus transmitted efficiently, but the other three viruses did not transmit in this animal model. We then pinpointed the key genetic determinants that contributed to the difference in transmissibilities of these H7N9 viruses and explored the underlying mechanism.

## RESULTS

### Transmissibilities of different H7N9 viruses in guinea pigs.

**FIG 1:**
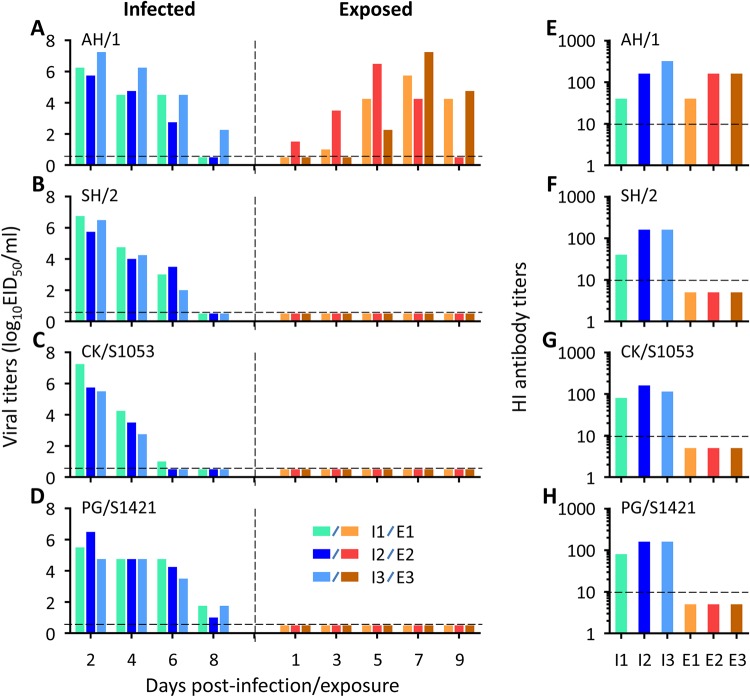
Respiratory droplet transmission of wild-type H7N9 viruses in guinea pigs. Groups of three guinea pigs were infected with 10^6^ EID_50_ of virus in a volume of 300 μl. Another three naive guinea pigs were placed in adjacent cages 24 h p.i. Nasal washes were collected at the indicated times and titrated in embryonated eggs. Seroconversion of the infected or exposed animals was tested for on day 21 p.i. or p.e. The black dashed lines indicate the titer detection limit. Each color bar represents the virus titer (A to D) or antibody titer (E to H) from an individual animal.

The four H7N9 viruses we tested in this study include two human isolates (AH/1 and A/Shanghai/2/2013 [SH/2]) and two avian isolates (CK/S1053 and A/pigeon/Shanghai/S1421/2013 [PG/S1421]). Three guinea pigs in three separate cages were intranasally infected with 10^6^ 50% egg infective doses (EID_50_) of the desired viruses in a volume of 300 μl, and 24 h after infection, three naive guinea pigs were put into three adjacent cages. Each pair of animals was separated by a double-layered net divider. Nasal washes were collected at 2-day intervals, beginning on day 2 postinfection (p.i.) from the infected animals or day 1 postexposure (p.e.) from the exposed animals, and titrated in embryonated chicken eggs. As shown in Fig. 1, AH/1 transmitted efficiently to all three guinea pigs, and one exposed guinea pig was infected as early as 1 day p.e. (Fig. 1A); the other three H7N9 viruses were not detected in the exposed guinea pigs (Fig. 1B to D). All of the guinea pigs that were infected with or exposed to the AH/1 virus seroconverted (Fig. 1E). The guinea pigs infected with the other three viruses seroconverted, but the exposed animals did not seroconvert (Fig. 1F to H). These results demonstrate that the H7N9 virus AH/1 transmits efficiently in guinea pigs via respiratory droplets but that the other three viruses do not transmit in this animal model.

### Transmissibilities in guinea pigs of different reassortants and mutants derived from the AH/1 backbone.

**FIG 2:**
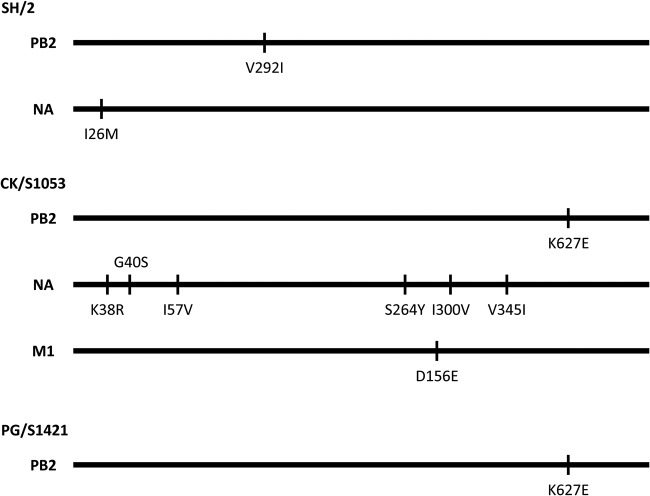
Amino acid differences between H7N9 viruses and the human isolate A/Anhui/1/2013 (AH/1). The amino acids are shown as single letters at the indicated positions. Each amino acid of the AH/1 virus is shown before the number of the position, and each amino acid of the other viruses is shown after the number of the position.

The H7N9 viruses isolated from poultry and humans in 2013 are genetically similar; the AH/1 virus differs by only one, two, and eight amino acids from the PG/S1421, SH/2, and CK/S1053 viruses, respectively (Fig. 2). We therefore use the “loss-of-function” strategy to explore which of these amino acids limits the transmission of H7N9 virus in guinea pigs. We rescued the AH/1 virus using plasmid-based reverse genetics as described by Li et al. (36) and designated the virus rAH/1. We then generated one reassortant that carries the NA gene of CK/S1053 virus and four single amino acid mutants in the AH/1 background; these viruses were designated rAH/1-CK/S1053-NA, rAH/1-PB2-292I, rAH/1-PB2-627E, rAH/1-NA-26M, and rAH/1-M1-156E. The transmissibility of the recombinant viruses was then tested in guinea pigs.

**FIG 3:**
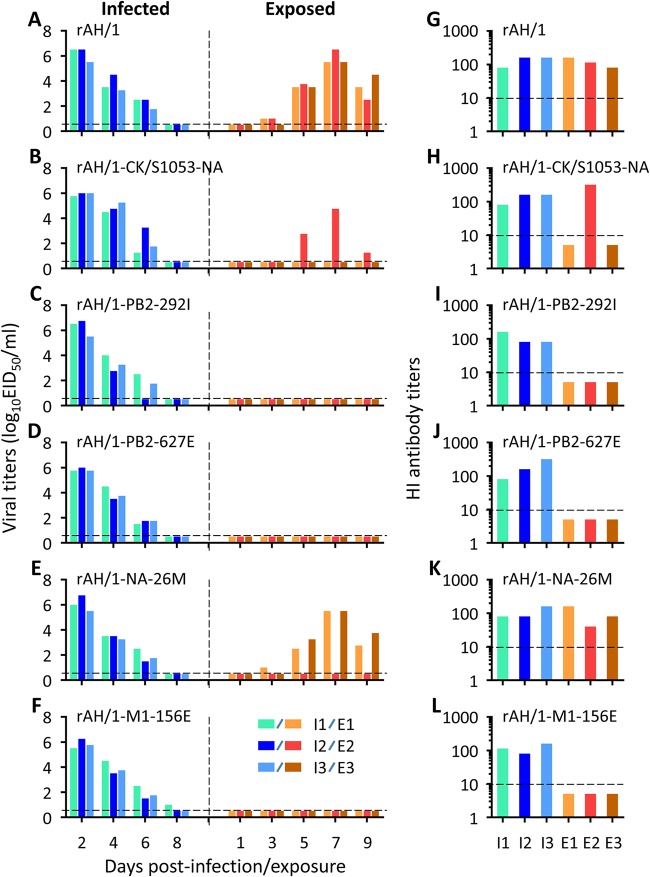
Respiratory droplet transmission of H7N9 reassortants and mutants in guinea pigs. Groups of three guinea pigs were infected with 10^6^ EID_50_ of virus in a volume of 300 μl. Another three naive guinea pigs were placed in adjacent cages 24 h p.i. Nasal washes were collected at the indicated times and titrated in embryonated eggs. Seroconversion of the infected or exposed animals was tested for on day 21 p.i. or p.e. The black dashed lines indicate the titer detection limit. Each color bar represents the virus titer (A to F) or antibody titer (G to L) from an individual animal.

Similar to wild-type AH/1, rAH/1 transmitted to all three exposed guinea pigs via respiratory droplets (Fig. 3A), and all of the infected and exposed animals in the rAH/1 group seroconverted (Fig. 3G). In the reassortant rAH/1-CK/S1053-NA group, virus was isolated from all three infected animals and one of the three exposed animals (Fig. 3B); seroconversion was detected in these virus-positive animals (Fig. 3H). In the mutant rAH/1-NA-26M group, virus was isolated from all three infected animals and two of the three exposed animals (Fig. 3E), and all of the infected and exposed animals seroconverted (Fig. 3K). In the rAH/1-PB2-292I, rAH/1-PB2-627E, and rAH/1-M1-156E groups, virus was isolated and seroconversion was detected in the infected animals but not in any of the exposed animals (Fig. 3C, D, F, I, J, and L). These results indicate that the mutation I26M in NA did not affect the transmissibility of the AH/1 virus, the NA gene of the CK/S1053 virus weakened the transmissibility of the AH/1 virus, and the mutations V292I and K627E in PB2 and D156E in M1 independently abolished the transmissibility of the AH/1 virus in guinea pigs. These results also suggest that the amino acids 292I and 627E of PB2 are the key factors that limited the transmission of SH/2 and PG/S1421, respectively, whereas 627E of PB2 and 156E of M1 are the key factors that limited the transmission of CK/S1053 virus.

### Replication of H7N9 wild-type viruses and mutants in human alveolar basal epithelial (A549) cells.

**FIG 4:**
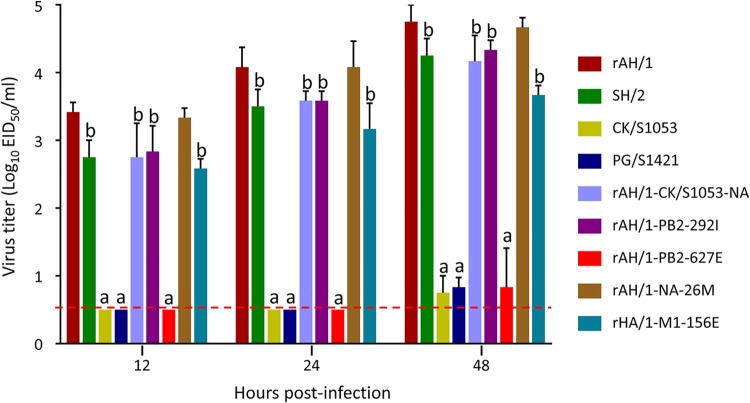
Growth kinetics of H7N9 viruses in A549 cells. A549 cells were infected with the test viruses at an MOI of 0.01 in triplicate. The supernatants were collected at the indicated time points for titration in eggs. The red dashed line indicates the lower limit of detection. Significance was analyzed by using one-way ANOVA with *post hoc* tests. a, *P < *0.001; b, *P < *0.05, compared with the corresponding value of the rAH/1 virus.

Replication efficacy is important for influenza virus transmission, so we compared the degrees of replication of rAH/1, SH/2, CK/S1053, PG/S1421, rAH/1-CK/S1053-NA, rAH/1-PB2-292I, rAH/1-PB2-627E, rAH/1-NA-26M, and rAH/1-M1-156E in A549 cells. As shown in Fig. 4, rAH/1-NA-26M replicated similarly to rAH/1 in A549 cells. The SH/2, rAH/1-CK/S1053-NA, rAH/1-PB2-292I, and rAH/1-M1-156E viruses replicated efficiently in A549 cells, but their titers were significantly lower than that of the rAH/1 virus (*P < *0.05). The CK/S1053, PG/S1421, and rAH/1-PB2-627E viruses, which do not have 627K in their PB2 proteins, replicated poorly and were not detected in the cells at 12 h or 24 h p.i.; the titers of these three viruses at 48 h p.i. were much lower than that of the rAH/1 virus (*P < *0.001) (Fig. 4). These data indicate that the replication efficacy of influenza viruses is affected by multiple factors.

### Role of mutations in the PB2 protein on the polymerase activity of H7N9 virus.

**FIG 5:**
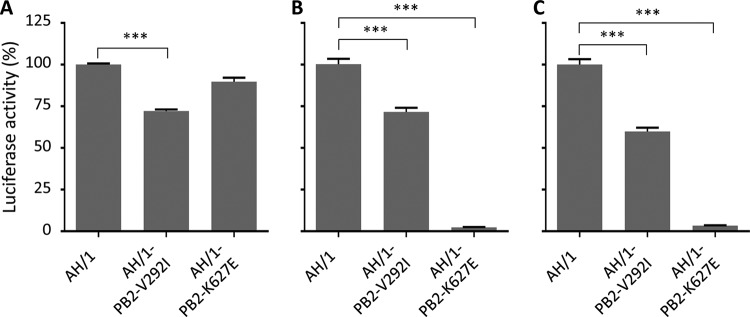
Polymerase activity of the RNP complex bearing different mutations in PB2. The polymerase activity was analyzed in DF-1 cells at 37°C (A) and in 293T cells at both 37°C (B) and 33°C (C). Cells were transfected with 0.25 μg each of pPol-Luc and the *Renilla* luciferase-expressing plasmid pRL-TK, together with PB2, PB1, PA, and NP expression plasmids. Cells extracts were harvested at 24 h posttransfection for the assay. Significance was analyzed by using one-way ANOVA with *post hoc* tests. ***, *P < *0.001.

The PB2 protein is a component of the viral ribonucleoprotein (RNP) complex, which plays a critical role in the transcription and replication of influenza A virus. Previous studies have reported that two amino acid mutations in PB2, E627K and D701N, increase the polymerase activities and thereby increase the virulence and transmissibility of different influenza viruses (22, 25, 26, 28, 37, 38). Our transmission study indicated that the mutations V292I and K627E of PB2 independently abolished the transmission of the AH/1 virus; therefore, we investigated how these two mutations affect the polymerase activities of the RNP complex of the AH/1 virus in different cells at 33°C and/or 37°C. As shown in Fig. 5, the mutation V292I in PB2 significantly reduced the polymerase activity of the RNP complex of the AH/1 virus in both chicken DF-1 fibroblasts (Fig. 5A) and human embryonic kidney 293T cells (Fig. 5B and C); K627E in PB2 also significantly reduced the polymerase activity of the RNP complex of the AH/1 virus in 293T cells (Fig. 5B and C) but not in DF-1 cells (Fig. 5A). These results indicate that the amino acids at positions 292 and 627 of PB2 affect the polymerase activity of the RNP complex of the H7N9 virus; the PB2 V292I mutation weakened the polymerase activity in both avian and mammalian cells, whereas the PB2 K627E mutation impaired the polymerase activity only in mammalian cells.

### NA activities of different H7N9 viruses.

**FIG 6:**
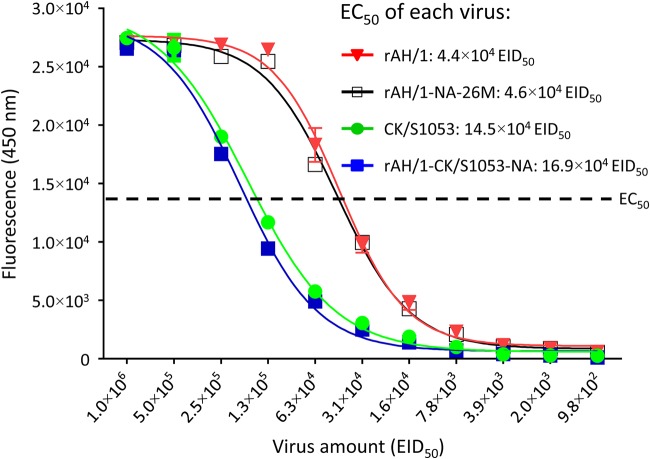
Neuraminidase activities of different H7N9 viruses. Neuraminidase activity was analyzed in the presence of the substrate MUNANA. The reaction was analyzed at excitation and emission wavelengths of 365 nm and 450 nm, respectively. The reactions were performed in triplicate three times. The dashed line shows the half-maximal effective concentration (EC_50_).

The NA protein promotes virus release by cleaving the sialic acids on the cell surface (39–42). A previous study by Lakdawala et al. reported that NA activity correlates with the release of virus particles and that increased viral release is important for efficient respiratory droplet transmission of the 2009/H1N1 virus (43). Our transmission study indicated that the NA gene of CK/S1053 reduced the transmissibility of the AH/1 virus in guinea pigs. Accordingly, we investigated whether the NA of CK/S1053 and AH/1 have different enzymatic activities; we also included rAH/1-NA-26M in this assay. As shown in Fig. 6, the enzymatic activities of the rAH/1 virus and rAH/1-NA-26M were similar but higher than those of the rAH/1-CK/S1053-NA and CK/S1053 viruses, and the half-maximal effective concentrations (EC_50_ values) of rAH/1, rAH/1-NA-26M, CK/S1053, and rAH/1-CK/S1053-NA were 4.4 × 10^4^ EID_50_, 4.6 × 10^4^ EID_50_, 14.5 × 10^4^ EID_50_, and 16.9 × 10^4^ EID_50_, respectively (Fig. 6). These results suggest that the relatively low enzymatic activity of the NA of the CK/S1053 virus may have contributed to the decreased transmissibility of the rAH/1-CK/S1053-NA virus.

### Morphology of H7N9 viruses.

**FIG 7:**
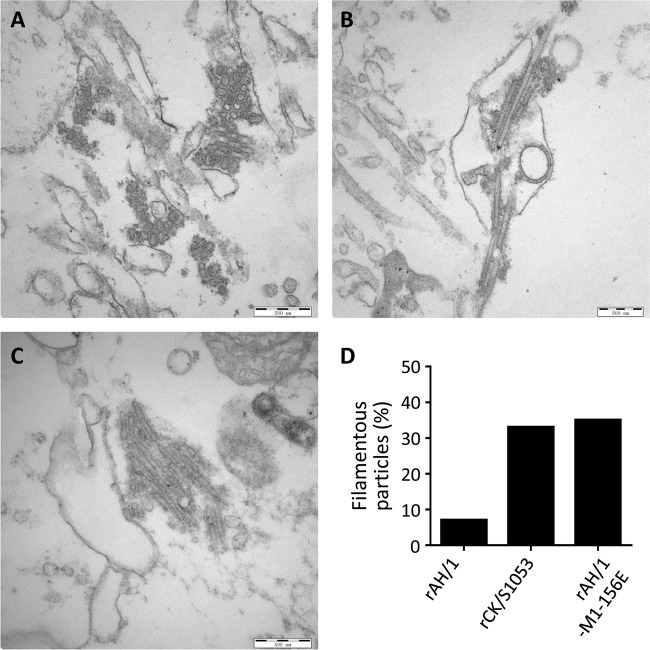
Morphology of H7N9 viruses. Typical electron micrographs of negatively stained viruses are shown. A549 cells were infected with rAH/1 (A), CK/S1053 (B), and rAH/1-M1-156E (C) at an MOI of 5 and fixed at 16 h p.i. (D) The filament and sphere ratios were calculated by counting 100 virus particles randomly. Filaments were defined as being at least 200 nm in length.

Influenza virus is pleomorphic, with particles exhibiting spherical or filamentous morphology, and the M1 protein is critical in determining the morphology of influenza viruses (44–47). Several studies have reported a correlation between a high ratio of ﬁlamentous viral particles and efﬁciency of transmission of H1N1 influenza viruses (43, 48–50). Our transmission study indicated that the D156E mutation in M1 abolished the transmissibility of the AH/1 virus in guinea pigs. We therefore investigated the morphology of the rAH/1, CK/S1053, and rAH/1-M1-156E viruses by using transmission electron microscopy. As shown in Fig. 7, spherical morphology was predominantly observed in the three viral samples (Fig. 7A to C). We then counted about 100 particles of each viral sample and found that the filamentous particles in the rAH/1 virus samples represented 7% of the total, whereas the filamentous particles in the CK/S1053 and rAH/1-M1-156E samples represented 33% and 35%, respectively (Fig. 7D). These results indicate that the AH/1 virus has a lower ratio of filamentous particles than the CK/S1053 virus, which we attribute to the amino acid difference at position 156 in M1.

### Prevalence of PB2 292V and M1 156D in H7N9 avian influenza viruses.

**FIG 8:**
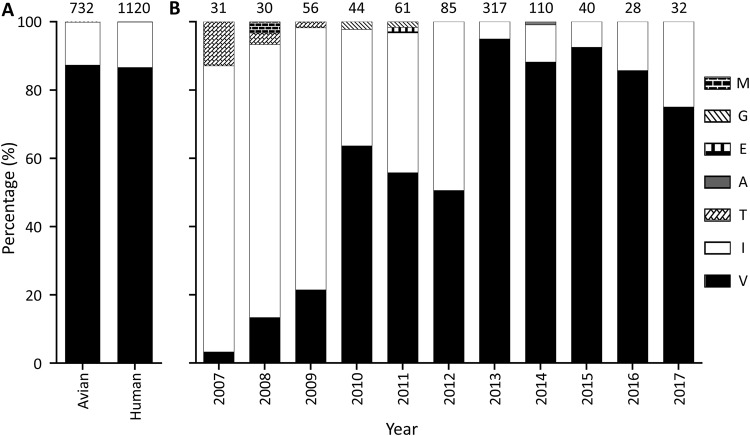
Amino acids at position 292 in the PB2 proteins of naturally isolated H7N9 and H9N2 inﬂuenza viruses. (A) H7N9 viruses. (B) H9N2 viruses. The number at the top of each bar shows the total number of strains analyzed. The sequence data of these strains were download from the National Center for Biotechnology Information (GenBank).

To investigate the prevalence of the two amino acids PB2 292V and M1 156D, which are important for H7N9 virus transmission, we analyzed the publicly available sequences of the H7N9 viruses. As shown in Fig. 8, about 87% of H7N9 viruses isolated from birds and humans already have the amino acid 292V in their PB2 proteins (Fig. 8A). Since the internal genes of H7N9 viruses were derived from H9N2 avian influenza viruses, we also analyzed the amino acid at position 292 in the PB2 proteins of H9N2 viruses that were detected from 2007 to 2017. We found that I or V at position 292 in PB2 is common in H9N2 viruses, but the proportion of H9N2 viruses bearing 292V in PB2 has gradually increased over the years (Fig. 8B). Analysis of the M1 sequences available in GenBank revealed that M1 156D is highly conserved among H7N9 and H9N2 viruses and that only two H7N9 viruses (A/chicken/Shanghai/S1053/2013 and A/chicken/Shanghai/S1055/2013) and two H9N2 viruses (A/chicken/Shandong/zc2/2009 and A/environment/Zhongshan/ZS201602/2016) bear 156M in their M1 proteins. These data indicate that the newly identified transmission-related mutations PB2 292V and M1 156D are already common among H7N9 viruses.

## DISCUSSION

In the present study, we compared the respiratory droplet transmissibilities of four H7N9 viruses in guinea pigs and found that the AH/1 virus is efficiently transmitted, but the other three viruses, SH/2, CK/S1053, and PG/S1421, are not transmissible in guinea pigs. Using a loss-of-function strategy, we found that low NA enzymatic activity impaired the transmission of H7N9 virus and that three amino acid mutations—V292I and K627E in PB2 and D156E in M1—independently abolished the transmission of the AH/1 virus. In other words, one or two of the amino acids at positions 292 and 627 of PB2 and the amino acid at position of 156 of M1 are the key factors that contribute to the different transmission phenotypes of AH/1 and the other three viruses. Although the PB2 E627K mutation has been reported to affect the transmission of different influenza viruses in ferrets and guinea pigs (26, 28), this is the first report that the amino acids 292V of PB2 and 156D of M1 are important for influenza virus transmission. Our findings thus provide new insights into the transmissibility of inﬂuenza virus.

Ferrets and guinea pigs are two important models that have been widely used to evaluate influenza virus transmission. The H7N9 influenza virus AH/1 did not transmit in humans and displays variable transmission rates in ferrets; Watanabe et al. found that AH/1 transmitted in one of three pairs of ferrets (51), Belser et al. found that the AH/1 virus transmitted in two of six pairs of ferrets (52), and Richard et al. found that AH/1 transmitted in three of four pairs of ferrets (53). The four viruses we tested in guinea pigs in this study were also previously tested in ferrets by our group (29). The AH/1 virus transmitted efficiently in both models, and CK/S1053 did not transmit in either model. SH/2 and PG/S1421 did not transmit in guinea pigs, but each virus transmitted in one of the three pairs of ferrets (29). Our findings indicate that efficiently transmissible and nontransmissible influenza viruses have the same transmission phenotype in these two models, whereas viruses with suboptimal transmissibility have different transmission patterns in these two animal models; that is, they may transmit in one model but not in the other. Therefore, the use of different animal models is helpful to fully understand the transmissibility of different inﬂuenza viruses.

Two amino acid mutations in PB2, E627K and D701N, have been previously reported to be important for the replication and transmission of different avian influenza viruses in mammals (25, 26, 28, 37, 38). Shi et al. reported that none of the H7N9 avian isolates bear the 627K or 701N mutation in the PB2 gene, but over 83% of H7N9 human isolates have these mutations (28), indicating that the E627K and D701N mutations in the PB2 protein of H7N9 human virus isolates are mammal-adapted mutations, although they have occasionally been detected in H5 influenza viruses isolated from avian species (36, 54). A recent study by Liang et al. reported that the emergence of the PB2 E627K mutation in H7N9 human influenza virus is driven by the intrinsic low polymerase activity conferred by the viral acid polymerase (PA) protein, which is also involved in the engagement of the mammalian ANP32A protein (55). The V292I mutation in PB2 abolished the transmission of the rAH/1 virus, even though this virus bears PB2 627K. Unlike the K627E mutation in PB2, which decreased the polymerase activity of the RNP complex of H7N9 virus in only mammalian cells and not avian cells, the V292I mutation decreased the polymerase activity of the RNP complex in both mammalian and avian cells. In other words, the I292V mutation in PB2 increases the polymerase activity of H7N9 viruses in both avian and mammalian cells. The amino acid 292V in the PB2 protein has been widely detected in H7N9 and H9N2 viruses. These findings indicate that I292V in PB2 is a host-independent mutation that promotes the transmission of H7N9 virus in mammals and facilitates the replication and spread of influenza viruses in avian species.

The neuraminidase activity of the influenza NA protein promotes virus release by cleaving sialic acids on the cell surface. Studies have shown that the NA protein of the 2009 pandemic H1N1 viruses, with higher neuraminidase activity than that of the classical swine NA, increased the release of virus particles and therefore improved the transmission of H1N1 viruses (43, 56, 57). Similar to these observations, we found that the rAH/1 virus had higher enzymatic activity than that of CK/S1053 and rAH/1-CK/S1053-NA, and the lower enzymatic activity of CK/S1053 NA was likely responsible for the lower replication in A549 cells and compromised the transmissibility of the rAH/1-CK/S1053-NA virus in guinea pigs. There are only six amino acid differences between the NA of AH/1 and that of CK/S1053, implying that only a few amino acid changes are responsible for the difference in enzymatic activity and transmissibility between rAH/1 and rAH/1-CK/S1053-NA.

The M1 protein is the most abundant protein in influenza virus particles and plays critical roles in the virus life cycle, including in virus entry, uncoating, assembly, and budding (10). In this study, we found that the amino acid 156D of M1 is critical for H7N9 virus transmission, since the single amino acid mutation of D156E in M1 abolished the transmission of the AH/1 virus in guinea pigs. The D156E mutation in M1 reduced the replication of the AH/1 virus in A549 cells and therefore reduced the transmissibility of the AH/1 virus in guinea pigs.

M1 is also an important determinant for the morphology of influenza virus (44–46). Previous studies reported that the Eurasian avian-like H1N1 swine influenza virus-origin M1 gene increased the ratio of filamentous particles of 2009 H1N1 pandemic virus and the laboratory-adapted A/Puerto Rico/8/1934 (H1N1) virus and thereby increased their transmissibility in mammals (43, 48). In the present study, we found that the filamentous particles of rAH/1 virus represented only 7% of the total virus particle population, yet the virus transmitted efficiently in guinea pigs. In contrast, filamentous particles of the CK/S1053 and rAH/1-M1-156E viruses represented about 33% and 35% of the total, respectively, yet these viruses did not transmit in guinea pigs. These data indicate that the amino acid at position 156 of M1 is important for determining the morphology of H7N9 influenza viruses and that a high ratio of filamentous particles is not a prerequisite for the H7N9 virus to transmit in animal models. These studies suggest that the morphologies of transmissible viruses may vary among different subtypes, and indeed, the cytoplasmic tails of both HA and NA have previously been shown to contribute to influenza viral morphology (58).

In summary, our study identified new key amino acids in PB2 and in M1 that play important roles in H7N9 inﬂuenza virus transmission. These findings further emphasize that the transmissibility of influenza virus is a polygenic trait, and efficient transmission of influenza virus relies on the different attributes and cooperation among the various viral proteins.

## MATERIALS AND METHODS

### Facility and ethics statements.

All experiments with live H7N9 viruses were conducted in a biosecurity level 3+ laboratory approved by the Ministry of Agriculture and Rural Affairs of the People’s Republic of China. All animals were used according to protocols approved by the Committee on the Ethics of Animal Experiments of the Harbin Veterinary Research Institute, Chinese Academy of Agricultural Sciences.

### Viruses.

Human H7N9 viruses AH/1 and SH/2 were kindly provided by Yuelong Shu, Chinese Center For Disease Control And Prevention, and the poultry H7N9 isolates CK/S1053 and PG/S1421 were isolated from live poultry markets (59). The mutants or reassortants were generated by using reverse genetics (60–62). To rescue the virus, viral RNA was extracted from allantoic fluid and the cDNAs of the eight gene segments were amplified and cloned into the vRNA-mRNA bidirectional expression plasmid pBD as described previously (36). Then 0.5 μg of each plasmid was transfected into 293T cells. Viruses in the cell supernatant were propagated in specific-pathogen-free eggs 48 h posttransfection. Each viral gene cDNA segment was sequenced to ensure the absence of unwanted mutations.

### Respiratory droplet transmission in guinea pigs.

Guinea pigs weighing 300 to 350 g (Vital River Laboratory, China) were used to evaluate the respiratory droplet transmission of the H7N9 viruses as described previously (63, 64). Briefly, three guinea pigs in three separate cages were intranasally infected with 10^6^ EID_50_ of the desired viruses in a volume of 300 μl, and 24 h after infection, three naive guinea pigs were put into the three adjacent cages. Each pair of animals was separated by a double-layered net divider. Nasal washes were collected four times from the infected animals and five times from the exposed animals at 2-day intervals, beginning on day 2 postinfection (p.i.) or day 1 postexposure (p.e.), and titrated in embryonated chicken eggs.

### HI assay.

The hemagglutination inhibition (HI) assay was used to determine antibody levels in the infected animals. Sera were treated with receptor-destroying enzyme (Denka-Seiken, Japan) according to the protocol of the WHO, and the sera were then diluted 1:2 serially. After incubating 25 μl of serum with 4 hemagglutinin units of virus in 25 μl, we added 50 μl of 0.5% (vol/vol) chicken red blood cells (RBCs) to the mixture. The highest dilution preventing the agglutination of the RBCs is the HI titer of the serum.

### Minigenome assay.

A dual-luciferase reporter assay system (Promega, USA) was used to calculate the activity of the RNP complex, which is composed of four proteins: PB2, PB1, PA, and NP. The genes were cloned into the expression vector pcDNA3.1. The reporter plasmid pPolI-Luc was constructed by inserting the open reading frame of the luciferase gene flanked by the 5′ and 3′ noncoding regions of the NP gene into a plasmid containing the human polymerase I promoter or the chicken polymerase I promoter. The RNP activity was analyzed in human 293T cells at 37°C and 33°C and in avian DF-1 cells at 37°C. Briefly, 0.25 μg each of pPol-Luc and the *Renilla* luciferase-expressing plasmid pTK-RL together with four pcDNA3.1 plasmids expressing PB2, PB1, PA, and NP protein were transfected into 293T cells or DF-1 cells. Cell extracts were harvested 24 h posttransfection, and the luciferase activity was assayed by using a 2014 multilabel plate reader (PerkinElmer, USA). The assay was performed in triplicate three times.

### Neuraminidase assay.

Neuraminidase activity was assayed in the presence of the substrate 2′-(4-methylumbelliferyl)-α-d-*N*-acetylneuraminic acid (MUNANA) (65, 66). The viruses were serially 2-fold diluted from 2 × 10^7^ EID_50_/ml, and the copy number of the M gene was quantitated by real-time PCR to ensure accurate virus dose as described by Yen et al. (56). Briefly, 50 μl of 200 μM MUNANA was mixed with 50 μl of virus dilution and incubated at 37°C for 10 min; the reaction was stopped by adding 100 μl of 1 M Na_2_CO_3_. The fluorescence was measured at excitation and emission wavelengths of 365 nm and 450 nm, respectively. The assay was performed in triplicate three times.

### Replication kinetics.

To evaluate the growth kinetics in human cells, A549 cells were infected with H7N9 virus at a multiplicity of infection (MOI) of 0.01 in triplicate at 37°C. One hour after infection, the cells were washed twice with phosphate-buffered saline. Then, Opti-MEM containing 0.1 μg/ml of tosyl phenylalanyl chloromethyl ketone trypsin was added to the cells. The supernatant was collected at 12, 24, and 48 h p.i. and titrated in 10-day-old chicken embryonated eggs.

### Virus morphology.

The morphology of rAH/1, CK/S1053, and rAH/1-M1-156E was observed by using transmission electron microscopy. Briefly, A549 cells were infected with the desired virus at an MOI of 5. The cells were then fixed with 2.5% (vol/vol) glutaraldehyde 16 h p.i. Ultrathin sections were prepared on carbon-coated 100-mesh copper grids and observed in a Hitachi-7650 transmission electron microscope at an operating voltage of 80 kV. Virus particles that were equal to or longer than 200 nm in length were defined as filamentous particles. The filamentous particle ratio was calculated by counting 100 virions randomly.

### Statistical analysis.

Significance was analyzed by using one-way analysis of variance (ANOVA) with *post hoc* tests. A *P* value of less than 0.05 was considered significant.
